# Touch and Go: Membrane Contact Sites Between Lipid Droplets and Other Organelles

**DOI:** 10.3389/fcell.2022.852021

**Published:** 2022-02-24

**Authors:** Pin-Chao Liao, Emily J. Yang, Taylor Borgman, Istvan R. Boldogh, Cierra N. Sing, Theresa C. Swayne, Liza A. Pon

**Affiliations:** ^1^ Department of Pathology and Cell Biology, Columbia University Irving Medical Center, New York, NY, United States; ^2^ Institute of Molecular Medicine, National Tsing Hua University, Hsinchu, Taiwan; ^3^ Institute of Human Nutrition, Columbia University Irving Medical Center, New York, NY, United States; ^4^ Herbert Irving Comprehensive Cancer Center, Columbia University Irving Medical Center, New York, NY, United States

**Keywords:** membrane contact sites, lipid droplets, mitochondria, endoplasmic reticulum, lysosome, vacuole, cytoskeleton, lipophagy

## Abstract

Lipid droplets (LDs) have emerged not just as storage sites for lipids but as central regulators of metabolism and organelle quality control. These critical functions are achieved, in part, at membrane contact sites (MCS) between LDs and other organelles. MCS are sites of transfer of cellular constituents to or from LDs for energy mobilization in response to nutrient limitations, as well as LD biogenesis, expansion and autophagy. Here, we describe recent findings on the mechanisms underlying the formation and function of MCS between LDs and mitochondria, ER and lysosomes/vacuoles and the role of the cytoskeleton in promoting LD MCS through its function in LD movement and distribution in response to environmental cues.

## 1 Introduction

Lipid droplets (LDs) have an established function in storing lipids, which are used for energy production, membrane biogenesis and synthesis of signaling molecules. LDs also function in storage of signaling proteins, their precursors and hydrophobic vitamins, and for sequestering toxic lipids, which is critical to reduce lipotoxicity and oxidative stress (Welte and Gould, 2017; Jarc and Petan, 2019; Geltinger et al., 2020; Roberts and Olzmann, 2020; Renne and Hariri, 2021). Finally, recent studies support a role for LDs in ER protein quality control (Garcia et al., 2018; Roberts and Olzmann, 2020).

The physical properties of LDs are distinct from those of other organelles. They consist of neutral lipids, primarily triacylglycerol (TAG) and sterol esters (SE), surrounded by a phospholipid monolayer. Although proteins are associated with LDs, conventional transport proteins that are integrated into lipid bilayers do not take part in transfer of lipids and other constituents from LDs to other organelles. Instead, specialized proteins, such as lipases that associate with the LD boundary membrane, release lipids and vitamin A from LDs (Schreiber et al., 2012; O'Byrne and Blaner, 2013; Grumet et al., 2016; Olzmann and Carvalho, 2019). Moreover, transfer of LD components to other organelles as well as communication between LDs and other subcellular compartments occurs at membrane contact sites (MCS) between LDs and other organelles.

MCS are sites of close apposition between two organelles. While these contacts may be homotypic (between identical organelles) or heterotypic (between different organelles), the focal point for this review article is heterotypic interactions between LDs and mitochondria, ER, lysosomes (the vacuole in yeast) and the role of the cytoskeleton in promoting contact site formation at LDs. LD MCS are not as well understood as other MCS. Nonetheless, LD MCS are enriched in proteins that mediate specific functions at those sites and are produced and stabilized by tethering proteins. Moreover, in yeast the distance between LDs and other organelles at MCS has been determined by electron microscopy to be <30 nm (Perktold et al., 2007; Binns et al., 2006), which is in the range of that observed in other MCS, typically 10–80 nm (Scorrano et al., 2019; Vance, 2020).

Although the structural components of many LD MCS have not been identified, the function of many LD MCS is well established. The endoplasmic reticulum (ER) constitutes the major site for the biogenesis of LDs and lipids that are incorporated into nascent LDs. Therefore, LD-ER contact sites are essential for LD formation, growth and budding from the ER (Olzmann and Carvalho, 2019; Choudhary and Schneiter, 2021). Recent studies revealed that LDs mediate removal of unfolded or damaged proteins from the ER, and that this occurs at LD-ER contact sites (Vevea et al., 2015; Garcia et al., 2021). At mitochondria, LDs deliver fatty acids, which are produced from neutral lipids that are stored in LDs and oxidized for energy production (Finn and Dice, 2006; Rambold et al., 2015; Wang et al., 2021). Toxic lipids or proteins that are sequestered in LDs can be delivered to lysosomes (the vacuole in yeast) by multiple pathways, including transfer events at LD-lysosome contact sites and piecemeal or wholesale uptake of LDs into the lysosome/vacuolar compartment (Tsuji et al., 2017; Schulze et al., 2020; Garcia et al., 2021; Liao et al., 2021). Finally, contacts between LDs and the cytoskeleton contribute to LD MCS formation through effects on LD movement and positional control (Pfisterer et al., 2017; Valm et al., 2017; Kilwein and Welte, 2019). Here, we review recent findings on the structure and function of LD MCS in yeast and mammalian cells, and how these membrane contacts respond to cellular or environmental cues.

## 2 LD Interactions With Mitochondria

Mitochondria are the metabolic centers of the cell. Fatty acids (FAs) that are stored as TAG and other lipids in LDs are used for energy production by β-oxidation in mitochondria. Conversely, mitochondria are the source of ATP and other components that contribute to growth or expansion of LDs. Close contacts between LDs and mitochondria were described in 1959 (Palade, 1959) and have been detected in many cell types (Novikoff et al., 1980; Stemberger et al., 1984). They are the sites for transfer of constituents between mitochondria and LD for LD consumption and expansion and are prominent in tissues with high energy demands such as heart (Kuramoto et al., 2012), skeletal muscle (Shaw et al., 2008), brown adipose tissue (Yu et al., 2015) and liver (Shiozaki et al., 2011; Ma et al., 2021). Although these contact sites have been evident for decades, recent studies have revealed important details of their function and structure.

### 2.1 LD-Mitochondria MCS Function in Transfer of Fatty Acids From LDs to Mitochondria

During periods of nutrient deprivation, cells reprogram their metabolism from glycolysis to oxidation of FAs for ATP production. During this process, FAs that are stored in TAG in LDs are transferred from LDs to mitochondria (Finn and Dice, 2006). Emerging evidence supports a role for LD-mitochondria MCS in this FA transfer event. First, starvation of cultured mammalian cells results in an increase in contact site formation between LDs and mitochondria (Herms et al., 2015; Rambold et al., 2015; Nguyen et al., 2017; Valm et al., 2017). Live-cell imaging of fluorescent FAs revealed that FAs move from LDs into mitochondria when nutrients are limiting. This process requires close association of mitochondria with LDs. It is also dependent on release of FA from TAG stored in LDs: depletion of an LD-associated neutral lipase, adipose triglyceride lipase (ATGL), or drug-induced inhibition of lipase activity reduces the mitochondrial accumulation of fluorescent FAs (Herms et al., 2015; Rambold et al., 2015; Valm et al., 2017).

**FIGURE 1:**
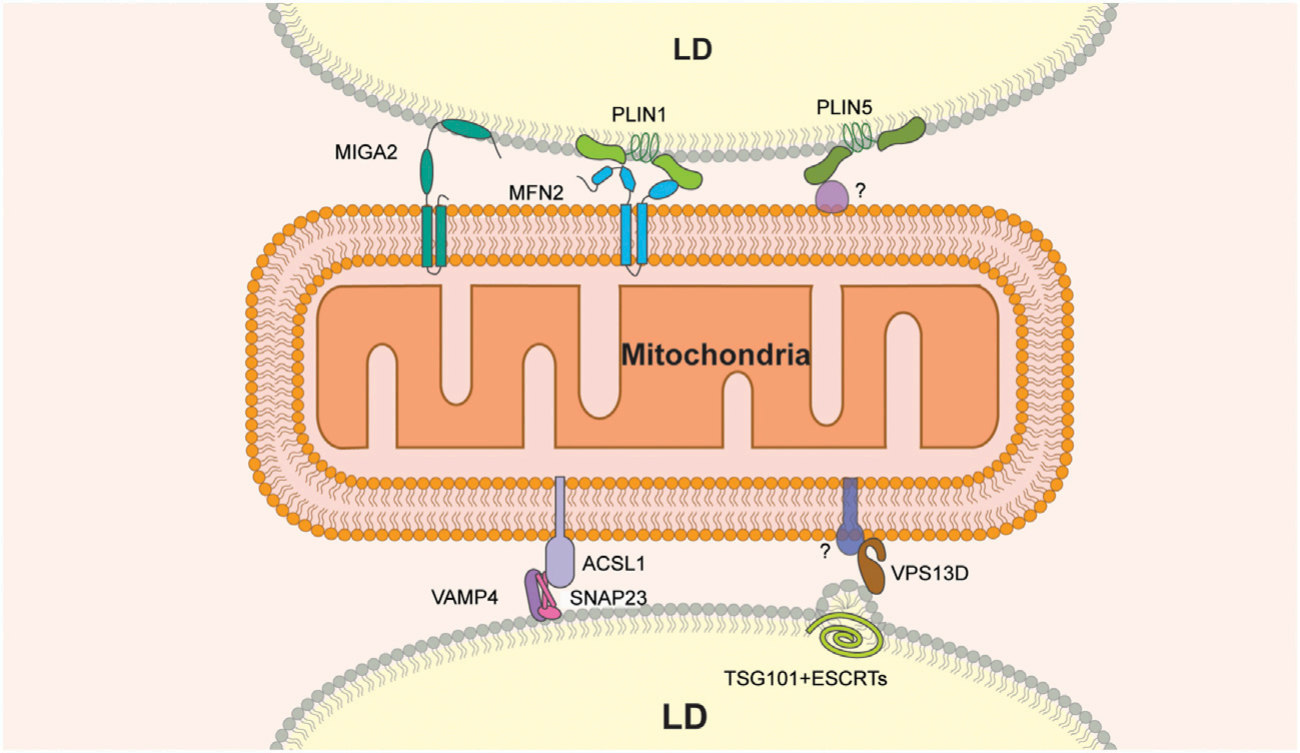
Molecular constituents of LD-mitochondria MCS. MIGA2 is the only LD-mitochondria tether that binds directly to both organelles. Other tethers include the VAMP4-SNAP2-ACSL1 or MFN2-PLIN1 protein complexes. VPS13D and PLIN5 are components of tethers that bind to LDs but have binding partners on mitochondria that have not been identified. In addition, VPS13D interacts with ESCRT on the LD surface and may contribute to membrane remodeling at that site.

Several proteins have been implicated in formation of these LD-mitochondria MCS (Figure 1). The SNARE proteins SNAP23 and VAMP4 localize to LDs in mouse fibroblasts (Boström et al., 2005), and SNAP23 has been detected on LDs and mitochondria in skeletal muscle (Strauss et al., 2016). More importantly, deletion of SNAP23 produces a decrease in both LD-mitochondria MCS and β-oxidation of radiolabeled FAs in mouse fibroblasts (Jägerström et al., 2009). A proximity labeling study revealed that ACSL1, a long-chain acyl-CoA synthetase that directs FAs to mitochondria for β-oxidation, interacts with SNAP23 and VAMP4 in hepatocytes (Young et al., 2018). In addition, glucose deprivation, a condition that stimulates FA oxidation, promotes co-immunoprecipitation of SNAP23, VAMP4 and ACSL1 in hepatocytes. (Young et al., 2018). These findings support the notion that increased association of LD and mitochondria contributes to elevated FA oxidation and indicate a role for SNAP23, VAMP4 and ACSL1 in establishing physical and functional interactions between LDs and mitochondria during this process.

Other studies support a role for the vacuolar protein sorting 13D (VPS13D) protein in FA transfer from LD to mitochondria at MCS between these organelles (Wang et al., 2021). VPS13D is a VPS13 family protein (Velayos-Baeza et al., 2004; Wang et al., 2021) that localizes to LD-mitochondria contact sites in response to oleic acid stimulation and starvation in cultured cells (Wang et al., 2021). Structure-function analysis revealed that the N-terminal region of VPS13D is responsible for mitochondrial targeting and that two amphipathic helices in the C-terminal region of the protein target VPS13D to the LDs. Moreover, VPS13D has a putative lipid transfer domain (LTD) at its N terminus that binds to FAs and is required for VPS13D function in FA transfer from LD to mitochondria. Finally, VPS13D recruits a subunit of the ESCRT (the endosome sorting complex required for transport), a complex that produces changes in membrane curvature (Vietri et al., 2020), to LD-mitochondria MCS. Specifically, the VAB (VPS13 adaptor binding) domain of VPS13D interacts with the ESCRT protein TSG101 and is required for recruitment of TSG101 to LD-mitochondria MCS. Moreover, localization of the VAB domain and TSG101 to this MCS results in the formation of a constricted or tubular structure at the surface of LDs (Wang et al., 2021). Finally, pulse-chase assays of FA transfer from LD to mitochondria revealed that the deletion of VPS13D or TSG101 results in a significant reduction of FA transfer (Wang et al., 2021). Collectively, these findings support a model for VPS13D in energy mobilization by FA oxidation in cells exposed to nutrient limitation. According to this model, VPS13D is recruited to LD-mitochondria junctions in response to starvation, where it contributes to FA transfer from LDs to mitochondria 1) as a lipid transfer protein and 2) by recruiting ESCRT components to LD-mitochondria MCS and facilitating ESCRT-dependent membrane remodeling at those sites.

Finally, the perilipin family protein perilipin 1 (PLIN1) has been implicated in LD-mitochondria contact site formation in brown adipose tissue through interactions with the mitochondrial outer membrane fusion GTPase, mitofusin 2, MFN2 (Boutant et al., 2017). MFN2 and its homolog MFN1 mediate the fusion of mitochondrial outer membranes. In addition, MFN2 is involved in mitochondria-ER contact sites (Giacomello et al., 2020). Nonetheless, depletion or knockout of MFN2 in brown adipose tissue results in fewer LD-mitochondria MCS, altered lipid metabolism and reduced FA oxidation by mitochondria (Boutant et al., 2017). In addition, co-immunoprecipitation studies show that MFN2 directly interacts with PLIN1, and this interaction is enhanced by a treatment with an adrenergic agonist. Finally, PLIN1 expression increases in mice subjected to cold treatment (Yu et al., 2015). These observations suggest that increased mitochondria-LD contacts mediated by MFN2-PLIN1 facilitate the coupling of TAG hydrolysis with FA oxidation upon exposure of brown adipose tissue to cold (Boutant et al., 2017).

### 2.2 LD-Mitochondria Contact Site Function in LD Expansion

Contact sites between LD and mitochondria can also function in expansion of LD under conditions that promote lipid storage. In brown adipose tissue, a subpopulation of mitochondria is closely associated with large LDs. Benador et al. (2018) developed a method to separate LD-associated mitochondria from LD-free mitochondria and found that these two populations of mitochondria are physically and functionally distinct. LD-associated mitochondria exhibit 1) elevated TCA cycle, ATP synthetic and pyruvate oxidation activities, 2) reduced β-oxidation activity, and 3) increased incorporation of free FAs into TAG in ATP synthase-dependent processes. Thus, contact site formation between LD and mitochondria is associated with lipid storage and generation of energy for this process by oxidation of glucose, not FAs. In contrast, LD-free mitochondria display higher FA oxidation. These observations support the idea that LD-associated mitochondria promote LD expansion and lipid storage by providing ATP for acyl-CoA synthesis during TAG production (Benador et al., 2018).

The LD protein perilipin 5 (PLIN5) has been implicated in LD-mitochondria interactions during LD expansion. PLIN5 is highly expressed in oxidative tissues, such as skeletal and cardiac muscle, brown adipose tissue and liver (Wolins et al., 2006), and is upregulated in response to exercise in muscle tissue (Tarnopolsky et al., 2007). Moreover, PLIN5 overexpression increases the number of LDs and the incorporation of radiolabeled lipids into TAG in brown adipose tissue and in cultured liver cells (Wang et al., 2011b; Benador et al., 2018). On the other hand, deletion of PLIN5 in mice results in a loss of LDs, and cultured cardiomyocytes from Plin5-null mice exhibit more FA oxidation activity compared to cardiomyocytes from wild-type mice (Kuramoto et al., 2012). Other studies indicate that PLIN5 function in LD expansion may be due to its function in LD-mitochondria MCS. PLIN5 can localize to the mitochondrial surface independent of LD-mitochondria MCS, and localizes to LD-mitochondria interfaces by super-resolution imaging (Gemmink et al., 2018). Moreover, overexpression of PLIN5 in CHO cells induces the recruitment of mitochondria to LD, and this recruitment depends on the presence of 20 amino acids at the C-terminal of the protein (Wang et al., 2011b). This observation supports the notion that PLIN5 is part of a tethering complex that promotes LD expansion at LD-mitochondria MCS.

Interestingly, in hepatocyte-specific Plin5 null mice, the decreased LD-mitochondria interactions resulted in reduced fatty acid oxidation and reduced fatty acid storage into TAGs (Keenan et al., 2019). Therefore, it is possible that even in tissues where PLIN5 is highly expressed, it can promote different aspects of LD-mitochondria interactions. Moreover, PLIN5 has been detected at mitochondria and in the cytoplasm independently of LD (Bosma et al., 2012; Gemmink et al., 2018), suggesting that it may also regulate lipid metabolism. Indeed PLIN5 also regulates the lipolytic activity of ATGL (Granneman et al., 2011; Wang et al., 2011a). These findings raise the possibility that PLIN5 affects TAG production via its regulatory activities on lipolysis independently from its mitochondrial tethering activity.

Mitoguardin 2 (MIGA2) is a mitochondrial outer membrane protein that promotes mitochondrial fusion and modulates body fat in mice by regulating mitochondrial phospholipid metabolism (Zhang et al., 2016). MIGA2 has also been implicated in LD-mitochondria MCS formation in differentiating white adipocytes (Freyre et al., 2019). Overexpression of MIGA2 in adipocytes leads to increased LD-mitochondria MCS formation (Freyre et al., 2019). Structure-function analysis of MIGA2 revealed a direct role for the protein in these MCS: its N-terminal transmembrane domains bind to mitochondria and its C-terminal amphipathic region is exposed to the cytosol and binds directly to LDs (Freyre et al., 2019). Finally, pre-adipocytes lacking MIGA2 exhibit reduced adipocyte differentiation, decreased LD abundance, and diminished TAG synthesis. Consistent with this, radiolabeled glucose is not converted into TAGs in MIGA2-knockout pre-adipocytes (Freyre et al., 2019). Collectively, these data suggest that MIGA2 is a tether that links LDs to mitochondria and raise the possibility that MIGA2 affects LD expansion through effects on *de novo* lipogenesis at MCS in adipocytes.

## 3 LD-ER Contact Sites

LDs form at and bud from the ER in all eukaryotes. LD biogenesis sites are the most complex and best characterized LD MCS. These MCS develop at specialized domains within the ER membrane, are enriched in specific lipids and proteins, and have a well-defined function in LD formation, directional growth and budding. These LD-ER MCS have activities found in other MCS including transfer of lipids and proteins between organelles. However, unlike other MCS in which a pre-existing organelle makes contacts with and is tethered to another organelle, LD-ER MCS develop within the ER membrane during LD biogenesis. While other MCS involve transitory interactions between two physically separate structures, the ER-LD MCS is not so simple. LDs and ER have different membrane and protein composition and different functional characteristics, but the distinction between these two compartments is less stark than, for example, that between ER and mitochondria. There is evidence from electron microscopy (Kassan et al., 2013) and fluorescence imaging (Jacquier et al., 2011; Valm et al., 2017) that in yeast, LDs and ER maintain long-term continuity. Fluorescence and biochemical studies in fly (Wilfling et al., 2013) and mammalian (Zehmer et al., 2009) cells have supported this model, although there are differences among cell types (Hugenroth and Bohnert, 2020).

Here, we describe formation of LD-ER contact sites, their function in LD biogenesis and the environmental cues that modulate these processes.

### 3.1 Formation of LD-ER MCS at Sites of LD Biogenesis

In light of the critical function of LDs in lipid storage and homeostasis, it is not surprising that LD biogenesis is regulated in response to changes in nutrient availability. Indeed, LD biogenesis is induced by nutrient limitations including the transition from mid-log to stationary phase in yeast, or nitrogen starvation (Jacob, 1987; Kurat et al., 2006; Li et al., 2015). It is also induced by supplementation with oleic acid (Callies et al., 1993; Fujimoto et al., 2006). In contrast, LD biogenesis is required for the survival of nutrient-limited cells (Sandager et al., 2002; Garbarino et al., 2009). One critical step in LD-ER contact site formation during LD synthesis is coalescence of neutral lipids (NL) to form a lens-shaped structure between the leaflets of the ER lipid bilayer. When the NL TAG reaches a threshold concentration (3–5 mol%), it undergoes a phase separation within the ER membrane leading to formation of the TAG lens (Khandelia et al., 2010; Duelund et al., 2013). In yeast, where these structures were first identified, NL lenses are ca. 50 nm in diameter (Choudhary et al., 2015).

**FIGURE 2:**
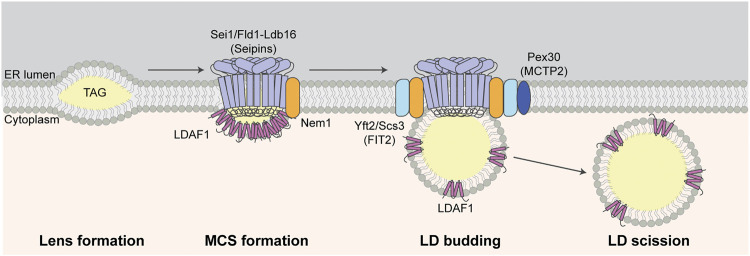
LD biogenesis at LD-ER MCS. TAG accumulates between leaflets of the ER bilayer during lens formation. Seipins, Nem1, and LDAF1 localize to and are required for LD-ER MCS formation at sites of LD biogenesis. Other LD biogenesis proteins including FITM2 and Pex30 are recruited to LD-ER MCS and LDAF1 is later transferred from MD-ER MCS to the surface of LDs during LD budding from the ER membrane. Finally, LDs are separated from ER and released to cytosol during LD scission.

The major molecular components and processes in LD-ER biogenesis are illustrated in Figure 2. Lens formation is induced by and requires synthesis of TAG and sterol esters (SE). In yeast, TAG is generated by acylation of the precursor diacylglycerol (DAG) by the diacylglycerol acyltransferases Dga1 and Lro1 (Lecithin cholesterol acyl transferase Related Open reading frame 1). SE are generated from sterols by the acyl-CoA:sterol acyltransferases Are1 and Are2. Indeed, inhibition of NL synthesis by deletion of all SE and TAG biosynthetic enzymes (*DGA1*, *LRO1*, *ARE1* and *ARE2*) blocks LD biogenesis (Sandager et al., 2002). Similarly, inhibition of DAG synthesis from phosphatidic acid by deletion of lipin (Pah1, phosphatidic acid phosphohydrolase 1, in yeast) results in reduced LD abundance (Adeyo et al., 2011).

The seipin protein complex determines the site of lens formation, mediates MCS formation between LDs and ER at those sites, and promotes TAG incorporation into lenses and nascent LDs. Seipin is encoded by the *BSCL2* (Berardinelli-Seip Congenital Lipodystrophy 2) gene in humans and *SEI1*/*FLD1* (Seipin/Few LDs) gene in yeast. It is an integral ER membrane protein that localizes to LD-ER contact sites (Szymanski et al., 2007; Fei et al., 2008; Salo et al., 2016; Wang et al., 2016). Seipin contains a highly conserved ER lumen domain, short N- and C-terminal cytosolic domains and two transmembrane domains (Lundin et al., 2006). The luminal domain contains a hydrophobic helix (HH) near the ER bilayer and a β-sandwich fold (Sui et al., 2018; Yan et al., 2018). The β-sandwich fold binds anionic phospholipids such as phosphatidic acid (Yan et al., 2018) and is similar in structure to β-sandwich domains in the sterol-binding Niemann-Pick C2 (NPC2) proteins. Recent, cryo-EM studies revealed that seipin oligomerizes to form a ring-like structure containing 10–12 subunits and that luminal HHs in that ring-like structure bind to TAG, which promotes TAG cluster formation at low concentrations (Prasanna et al., 2021; Zoni et al., 2021). Interestingly, yeast seipin lacks the HH domain found in human or *Drosophila* seipins. However, yeast seipin binds to Ldb16 (low dye binding 16), which contains HH-like regions and supports HH function in the yeast seipin complex (Klug et al., 2021).

Seipin functions in LD-ER MCS and LD formation through its interactions not just with lipids but with proteins including Nem1 (nuclear envelope morphology 1) and LDAF1 (LD activator factor 1), also known as Tmem159 and promethin in mammals, and Ldo45 (LD organization 45 kD protein) in yeast. Seipin-Nem1 interactions promote NL biosynthesis at sites of lens formation. Both proteins localize to and co-localize at punctate structures at sites of lens formation and do so independent of NL biosynthesis or the presence of LDs (Choudhary et al., 2020). Nem1 activates DAG production, and functions with seipin to recruit TAG biosynthetic enzymes (Dga1 and Lro1) at LD-ER MCS during lens initiation and growth (Choudhary et al., 2020).

Interaction of seipin with LDAF1 is also critical for the TAG phase transition during initiation of lens formation. Although small lens-likes structures can form in the ER membrane in the absence of seipin (Salo et al., 2016; Wang et al., 2016), recent studies support the model that seipin and LDAF1 stimulate lens formation by lowering the critical concentration of TAG for phase conversion within membranes. Specifically, deletion of LDAF1 inhibits LD formation during early stages of that process at all TAG concentrations tested, indicating that LDAF1 is required for initiation of LD biogenesis. Notably, it is released from seipin and recruited to the surface of nascent LDs as they mature (Chung et al., 2019). Consistent with this, molecular simulation studies revealed that binding of seipin to TAG promotes its association with LDAF1, which stabilizes nascent lens structures (Prasanna et al., 2021; Zoni et al., 2021). Finally, targeting of LDAF1 to the plasma membrane (PM) results in formation of PM-ER MCS, as well as recruitment of seipin and LD biogenesis at that site. Thus, seipin and LDAF1 can drive lens formation and LD biogenesis *in vivo* (Chung et al., 2019).

### 3.2 Generation of Lipid and Protein Asymmetry at LD-ER MCS During LD Growth and Budding

LD-ER interactions at sites of LD biogenesis are disrupted when nascent LDs bud from the ER into the cytosol. Budding of LDs from the ER and the size of LDs that are released from ER are influenced by membrane curvature and surface tension at the LD-ER MCS. Phospholipids that promote negative membrane curvature, such as DAG or phosphatidylethanolamine (PE), stabilize the LD-ER contact site and favor retention of LDs in the ER. In contrast, lysolipids, which promote positive membrane curvature, destabilize LD-ER MCS and favor LD budding (Choudhary et al., 2018) and generation of small LDs (Ben M’barek et al., 2017).

Fat storage-inducing transmembrane protein 2 (FITM2) is an evolutionarily conserved ER-localized transmembrane protein that is required for budding of LDs from ER membranes (Choudhary et al., 2015). Studies in yeast indicate that FITM2 proteins promote this process by regulating the levels of DAG. Although DAG is a precursor for TAG and therefore required for LD biogenesis, DAG can inhibit budding of nascent LDs from LD-ER MCS by promoting negative membrane curvature at those contact sites. Therefore, its levels must be tightly regulated during LD biogenesis. Indeed, lysolipids promote positive membrane curvature and budding of LDs from ER in the absence of FITM2 in yeast. This suggest that the increase of membrane curvature by lysolipids reduces the defects in LD biogenesis caused by high DAG levels in the absence of FITM2 (Choudhary et al., 2018). The FITM2 proteins of yeast (Yft2 and Scs3) are recruited to sites of LD biogenesis by binding to seipin and Nem1 (Choudhary et al., 2018; 2020). Moreover, deletion of both FITM2 proteins in yeast results in increased DAG and this defect is rescued by deletion of *NEM1* (Choudhary et al., 2018). Since Nem1 promotes DAG production, FITM2 proteins may modulate DAG levels though effects on Nem1.

Interactions between seipin and Pex30 (Peroxisome-related 30) have been implicated in modulation of the phospholipid composition at LD-ER MCS during lens formation. This process is downstream of the recruitment of FITM2 proteins to seipin-Nem1 sites (Choudhary et al., 2020). Pex30 is an ER membrane protein with established functions in control of peroxisome size, shape and formation (Joshi et al., 2016; Vizeacoumar et al., 2003; 2004). Interestingly, Pex30 is associated with seipin complexes at LD-ER contact sites during LD formation. Moreover, deletion of Pex30 results in abnormal LD morphology, and deletion of seipin and Pex30 results in inhibition of LD biogenesis, abnormal ER morphology, and growth defects (Joshi et al., 2018; Wang et al., 2018). Notably, the defect in LD biogenesis in *sei1∆ pex30∆* double mutants is rescued by deletion of Pct1 (phosphocholine cytidylyltransferase 1), the rate-limiting enzyme in the phosphatidylcholine (PC) biosynthesis Kennedy pathway. PC is the most abundant phospholipid in the LD membrane. Thus, Pex30 may contribute to LD biogenesis by modulating phospholipid composition in the LD-ER contact site and on the surface of the nascent LD during LD biogenesis (Wang et al., 2018). Interestingly, Pex30 contains membrane-shaping reticulon-like regions (Joshi et al., 2016) and may also contribute to deforming the membrane at LD-ER MCSs and budding of the nascent LDs from the ER membrane.

### 3.3 Role for ERAD in Removal of Surplus LD Proteins From the ER Membrane

The ER-associated degradation pathway (ERAD) was originally identified as a pathway for degradation of unfolded or damaged proteins in ER membranes. In ERAD, unfolded proteins are ubiquitinated, recognized and extracted by the AAA-ATPase Cdc48 in yeast (p97/VCP in mammals), and degraded by proteasomes (Christianson and Ye, 2014). Recent studies support a novel role for ERAD in degrading LD proteins within the ER membrane.

In mammals, diacylglycerol acyltransferase 2 (DGAT2), an enzyme that catalyzes the conversion of DAG to TAG, is degraded by ERAD with the aid of the ubiquitin ligases gp78 and Hrd1 (Choi et al., 2014; Luo et al., 2018). In yeast, a subset of LD proteins, Pgc1 (phosphatidyl glycerol phospholipase C), Dga1, and Yeh1 (yeast steryl ester hydrolase), are substrates for the ERAD ubiquitin ligase Doa10 and degraded by ERAD. The HH domain of Pgc1 has been implicated as a degron for ERAD: it is both necessary and sufficient for Doa10-dependent degradation (Ruggiano et al., 2016). Interestingly, degradation of Pgc1 by ERAD is accelerated in the absence of yeast FITM2 (Yap et al., 2020). Moreover, the regions for ERAD degradation and for targeting of proteins to LDs overlap (Ruggiano et al., 2016). These findings raise the possibility that proteins that are not incorporated into LDs are degraded in the ER by ERAD.

### 3.4 LD-ER Contact Sites and ER Proteostasis

**FIGURE 3:**
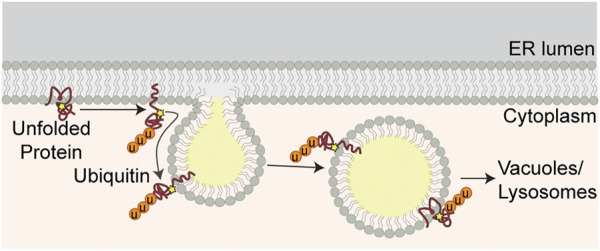
ER proteostasis at LD-ER MCS. Unfolded ER proteins are marked for degradation by ubiquitination. Ubiquitinated proteins are transferred from ER to LDs at LD-ER MCS. LDs containing ubiquitinated proteins bud from the ER and are delivered to vacuoles for degradation by microautophagy.

As described above, resident LD proteins are recruited to nascent LDs at LD-ER MCS. Recent evidence indicates that unfolded ER proteins, which accumulate in ER under conditions of ER stress and compromise ER and cellular function and fitness, are removed from the ER in LDs by transport from ER to LDs at LD-ER MCS. In contrast to the ERAD system which relieves ER stress by removing individual unfolded proteins from the organelle, this LD-based ER proteostasis mechanism enables high-throughput removal of unfolded ER proteins (Figure 3) (Vevea et al., 2015).

Early studies revealed that ER proteins are recovered in isolated LDs. Although these proteins were first interpreted as contaminants in LD preparations, several lines of evidence indicate that ER proteins are recruited to LDs by ER stress. Specifically, treatment of yeast with a reducing agent, dithiothreitol, which inhibits oxidative folding in the ER, results in recruitment of 1) proteins that contain disulfide linkages and undergo oxidative folding in the ER, 2) protein disulfide isomerase (PDI) proteins, multifunctional ER redox chaperones, and 3) other ER chaperones to LDs. Similarly, treatment with tunicamycin, an agent that induces protein misfolding by inhibiting protein glycosylation in the ER, results in recruitment of proteins that are glycosylated in ER and the ER chaperones described above to LDs. Imaging studies revealed that ER proteins that are recovered with LDs also co-localize with LDs in living yeast exposed to ER stress. These imaging studies also provide documentation of 1) association of LDs with protein aggregates in the ER membrane, 2) co-localization of those protein aggregates with LDs as they bud from ER membranes and move away from the ER, and 3) localization of LDs and their associated ER protein aggregates in the vacuole (yeast lysosome) (Garcia et al., 2021).

Equally important, LD function in ER protein quality control is a physiologically relevant stress response. Indeed, LD biogenesis or abundance is up-regulated in response to ER stressors in yeast (Fei et al., 2009; Vevea et al., 2015; Garcia et al., 2021), in mammalian cells (Lee et al., 2012) and in mouse liver (Yamamoto et al., 2010; Zhang et al., 2011). Furthermore, inhibition of LD biogenesis dramatically reduces cellular growth and survival in yeast challenged by ER stressors (Garcia et al., 2021). Overall, these studies support a model for LD function in ER protein quality control whereby unfolded proteins are transferred from ER membranes to nascent LDs at LD-ER MCS, removed from ER by LDs as they bud from the ER and degraded in response to ER stress.

## 4 LD-Lysosome/Vacuole MCS

The lysosome (vacuole in yeast) plays a major role in catabolism, recycling of cellular waste, excretion of waste products and cellular signaling. Contact site formation between LDs and lysosomes/vacuoles plays direct and indirect roles in LD autophagy (lipophagy). Lipophagy, in turn, is essential for the mobilization of LD-bound lipids for energy production in response to nutrient limitations and other stressors, and for degradation of excess or toxic lipids or unfolded proteins that are stored and sequestered in LDs during ER stress. Lipophagy is also critical for delivery of sterols and other lipids in LD to the vacuolar membrane in the stationary phase in yeast (Tsuji et al., 2017; Garcia et al., 2018; Jarc and Petan, 2019).

**FIGURE 4:**
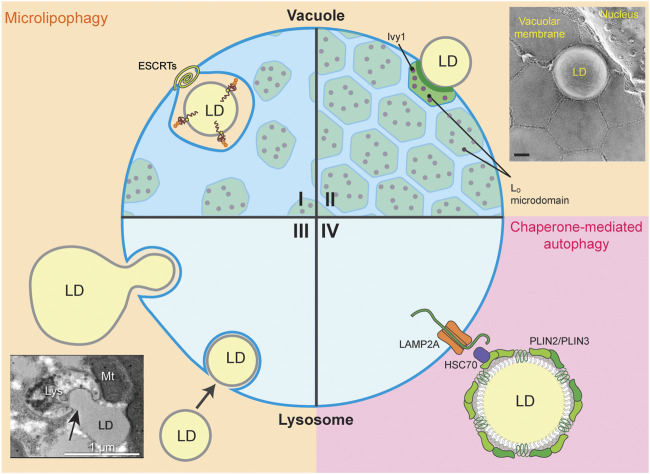
LD-Lysosome/Vacuole MCS. The four quadrants **(I-IV)** display the different types of LD-lysosome/vacuole MCS during microlipophagy (µLP, beige) and chaperone-mediated autophagy (CMA, pink). **(I, II)** MCS between LD and the yeast vacuole form during µLP. **(I)** The ESCRT machinery is required for µLP under DTT-, TM-, or lipid imbalance- induced ER stress and during diauxic shift. Ubiquitinated, unfolded proteins on LDs are engulfed by the vacuole for degradation. Small amounts of L_o_ microdomains associated with the Ivy1 protein appear in cells under ER stress. However, LDs are not taken up through these L_o_ microdomains. **(II)** L_o_ microdomain-dependent uptake of LDs is typical of cells in the stationary phase or under nitrogen starvation, and to some extent in cells under lipid imbalance. Ivy1-containing L_o_ microdomains are widespread under these conditions. Inset, freeze-fracture EM showing L_o_ microdomains during stationary phase-induced µLP (image from Tsuji et al. (2017) with permission, scale bar 0.2 µm). **(III, IV)** LD-lysosome MCS in mammalian cells are shown during µLP through partial (piecemeal) or whole-LD uptake and CMA. **(III)** µLP may occur via piecemeal uptake of a larger LD into the lysosome, or by wholesale uptake of smaller LDs into the lysosome. Inset, transmission electron micrograph of rat primary hepatocytes treated with oleic acid to induce LD formation and serum-starved HBSS to induce LD autophagy (image from Schulze et al. (2020) with permission, scale bar 1 µm). (IV) LD-associated proteins Plin2 and Plin3 are degraded via CMA in the lysosome. Hsc70 binds PLIN2 and PLIN3 and delivers these LD-associated proteins to LAMP2A to be translocated from the lysosomal surface to the lumen for degradation.

LD-lysosome/vacuole MCS have been implicated in three forms of lipophagy. In LD macroautophagy, which is the primary form of lipophagy in mammalian systems, LDs are encapsulated within autophagosomes, and delivered to the lumen of the lysosome by fusion of autophagosomes with the lysosomal membrane (Singh et al., 2009). In LD microautophagy or microlipophagy (µLP) which is predominantly understood in yeast, LDs make direct contact with the lysosome/vacuole and partial or wholesale uptake of LDs into the lysosome/vacuole at sites of invagination in the lysosome/vacuole membrane (Garcia et al., 2018; Schulze et al., 2020). Finally, in chaperone-mediated autophagy (CMA), specific LD proteins are targeted to the lysosome by chaperones and translocated across the lysosomal membrane by the lysosome-associated membrane protein type 2A (LAMP2A) (Kaushik and Cuervo, 2015; 2016). All three forms of autophagy are induced by nutrient limitation and other environmental cues. Below, we review the two forms of lipophagy that occur by direct contact between LDs and the lysosome/vacuole at MCS between the organelles: LD microlipophagy (Figures 4I–III) and CMA (Figure 4IV).

### 4.1 LD-Vacuole MCS During LD Microautophagy in Yeast

Microlipophagy (µLP) was first identified in yeast, and has emerged as the primary mechanism for lipophagy in yeast. µLP can be induced by stressors including nitrogen or glucose limitation, entry into stationary phase, lipid imbalance, and ER stress. Although these conditions all induce µLP, two forms of µLP occur at distinct LD-vacuole MCS and require distinct factors that modulate vacuolar membrane dynamics, invagination and scission (van Zutphen et al., 2014; Wang et al., 2014; Vevea et al., 2015; Oku et al., 2017; Seo et al., 2017; Tsuji et al., 2017; Garcia et al., 2021; Liao et al., 2021). Below, we describe these two mechanisms of µLP at LD-vacuole MCS in yeast and the role of specific proteins and lipids in that process.

#### 4.1.1 LD-Vacuole MCS at Lo Microdomains During µLP in Yeast

In µLP induced by entry into stationary phase or nitrogen starvation, LDs make contacts with the vacuole at liquid ordered (L_o_) microdomains in the vacuolar membrane (Tsuji et al., 2017; Wang et al., 2014) (Figure 4II). L_o_ microdomains are lipid raft-like regions that are enriched with sterols and have distinct protein and lipid composition compared to the bulk of the vacuolar membrane, which has been referred to as a liquid disordered (L_d_) domain. Transfer of sterols from LDs to vacuoles at LD-vacuole MCS during L_o_ microdomain formation in stationary-phase yeast cells (Wang et al., 2014) and intravacuolar transfer of sterols to L_o_ microdomains by Neiman-Pick proteins mediates formation of these microdomains under multiple stress conditions (Tsuji et al., 2017; Liao et al., 2021). These microdomains form in response to various stresses including entry into stationary phase, nitrogen or glucose starvation, osmotic stress, cycloheximide (CHX)-mediated translation inhibition, weak acids, heat, and ER stress induced by lipid imbalance, DTT, or TM (Toulmay and Prinz, 2013; Liao et al., 2021). Thus, Lo microdomain formation is a general stress response (Figures 4I, II).

Moreover, vacuolar membrane proteins are enriched in and excluded from vacuolar L_o_ microdomains. Vph1, a component of vacuolar proton pump ATPase, is excluded from L_o_ microdomains. In contrast, sterol transporters (LaM6/Ltn1 and Nce102), TORC1 (target of rapamycin complex 1) subunits (Tco89, Tor Complex I 89) and subunits or interactors of the TORC1-regulating EGO/ragulator complex (Ivy1, Interacting with Vps33 and Ypt7; Gtr1 and 2, GTP binding protein resemblance 1 and 2; Iml1, increased minichromosome loss 1) are enriched in L_o_ microdomains (Toulmay and Prinz, 2013; Wang et al., 2014; Murley et al., 2015, 2017; Numrich et al., 2015; Varlakhanova et al., 2018; Vaskovicova et al., 2020).

The mechanisms underlying LD MCS formation at L_o_ microdomains and the vacuolar membrane dynamics and invagination at those MCS during release of LDs into the vacuolar lumen are not well understood. However, Ivy1 can bind to Ypt7, the Rab7 GTPase of yeast, and requires Ypt7 for localization to invaginations in the vacuolar membrane in response to nutrient limitation (Lazar et al., 2002; Numrich et al., 2015). Moreover, as described below, Rab7 has been implicated in LD-lysosome MCS formation in mammalian cells (Schroeder et al., 2015). Thus, Ivy1 may contribute to µLP through effects on MCS formation between LDs and L_o_ microdomains on the vacuolar membrane. Interestingly, Ivy1 is also a phospholipid-binding protein that contains a putative I-BAR domain, which binds to and stabilizes membranes with negative membrane curvature (Itoh et al., 2016). Therefore, Ivy1 may contribute to the invagination of the vacuolar membrane at contact sites between LDs and vacuolar membrane L_o_ microdomains (Figure 4IV).

#### 4.1.2 Lo Microdomain-Independent, ESCRT-Dependent µLP in Yeast

µLP is induced by the diauxic shift from glycolysis to respiration-driven metabolism during late log phase in yeast (Oku et al., 2017). Moreover, in response to ER stress, LDs that contain unfolded ER proteins are targeted for degradation by µLP (Vevea et al., 2015; Garcia et al., 2021). Although many stressors induce L_o_ microdomain formation in the vacuolar membrane, LDs do not form MCS with the vacuole at L_o_ microdomains during µLP induced by ER stressors or the diauxic shift in yeast. Rather, under these conditions, LD-vacuole MCS form at L_d_ domains in the vacuolar membrane that contain Vph1, which is excluded from L_o_ microdomains (Vevea et al., 2015; Oku et al., 2017; Garcia et al., 2021). In addition, ESCRT complex proteins are up-regulated and recruited to sites of membrane scission at these LD-vacuole MCS, and are required for L_o_ microdomain-independent µLP in yeast (Vevea et al., 2015; Oku et al., 2017; Garcia et al., 2021) (Figure 4I).

The mechanisms underlying LD-vacuole MCS formation during ER stress-induced µLP are not well understood. However, recent studies indicate that ER stressors induce vacuolar fragmentation in yeast. Moreover, LDs develop persistent interactions with clusters of fragmented vacuoles during L_o_ microdomain-independent µLP, which supports MCS between LDs and one or more vacuoles during this process. The fragmented vacuoles fuse to form a cup-shaped structure surrounding LDs, and then engulf the LDs. ER stress-induced µLP is blocked by inhibition of this vacuolar fusion (Garcia et al., 2021). Overall, these studies show that vacuolar fragmentation, clustering and fusion around LDs occur during stress-induced µLP, but ongoing studies are needed to determine tmore of the components and regulators of the MCS involved in µLP. Additionally, it has been discovered that the deletion of Rab7, a protein implicated in LD-lysosome MCS, results in accumulation of enlarged, clustered lysosomal compartments (MVBs) in mammalian cells (Schroeder et al., 2015), so it is possible that the clustering and fusion of degradative compartments is a conserved component of the µLP pathway.

### 4.2 LD-Lysosome MCS During Microlipophagy (µLP) in Mammalian Cells

LD degradation by macroautophagy has been studied extensively in mammalian cells. However, LD microautophagy (µLP) also occurs in mammalian cells, as revealed in recent studies of nutrient limitation in hepatocytes (Schulze et al., 2020). These studies documented formation of MCS between LDs and lysosomes, and uptake of LD segments or of intact LDs into lysosomes at invaginations in the lysosome membrane. Specifically, live-cell visualization of pH-sensing mRFP1-GFP targeted to the LD marker protein PLIN2 revealed persistent (>60 s) interactions between LDs and lysosomes and uptake of LDs into the acidic lumen of the lysosome under nutrient-limited conditions (Figure 4III). Interestingly, nutrient limitation resulted in an increase in the frequency of persistent LD-lysosome contacts. Moreover, silencing of canonical macroautophagy or CMA components has no effect on persistent LD-lysosome contacts, and EM studies revealed that MCS formation between LDs and lysosomes occurs in the absence of double-membrane, autophagosome-like structures. These findings provide the first evidence that LD degradation in response to nutrient limitations can occur by µLP in mammalian cells (Schulze et al., 2020).

The mechanism underlying µLP in mammalian cells is not well understood. However, emerging evidence supports a role for Rab7, a small GTPase and important regulator of endocytic trafficking, in LD-lysosome MCS formation in hepatocytes (Schroeder et al., 2015). Specifically, nutrient limitations result in recruitment of Rab7 to LDs, and an increase in MCS between LDs and degradative compartments including lysosomes, MVBs and late endosomes. Moreover, depletion of Rab7, or inactivating mutation of Rab7, inhibits interactions of LDs and degradative compartments and results in an accumulation of enlarged, clustered MVBs and an overall inhibition of starvation-induced LD degradation. This raises the interesting possibility that Rab7 mediates contact site formation between LDs and lysosomes directly, or by promoting MCS formation between LDs and late endosomes/MVBs (amplisomes) and that late endosomes/MVBs at these MCS mature to form lysosomes (Schroeder et al., 2015). Interestingly, Rab7 has also been implicated in LD activities that may affect LD MCS through effects on vacuolar fusion or LD motility.

### 4.3 LD-Lysosome MCS During CMA in Mammalian Cells

Although CMA typically targets soluble cytosolic proteins, the LD-associated perilipin proteins PLIN2 and PLIN3 are degraded by CMA at LD-lysosome MCS in cultured mammalian cells. (Kaushik and Cuervo, 2015; 2016; 2018). PLIN2 functions in LD biogenesis, stability and trafficking and serves as a scaffold that regulates association of LDs with the macroautophagy machinery (Tsai et al., 2017). PLIN3 also regulates macroautophagy in a TORC1 (target of rapamycin 1) -dependent manner (Garcia-Macia et al., 2021). Starvation-induced CMA of PLIN2 and PLIN3 is mediated by the 70-kD heat shock protein, hsc70, which binds to the pentapeptide motifs LDRLQ on PLIN2 and SLKVQ on PLIN3, promotes phosphorylation of PLIN2 by 5′ AMP-activated protein kinase (AMPK), and delivers PLIN2 and PLIN3 to the lysosome-associated membrane protein 2A (LAMP2A) (Kaushik and Cuervo, 2015; 2016; 2018), the vacuolar membrane protein that facilitates translocation of CMA substrates from the lysosomal surface to the lumen (Chiang et al., 1989; Salvador et al., 2000; Bandyopadhyay and Cuervo, 2008). Deletion of the pentapeptide CMA recognition motif on PLIN2 results in an increase in PLIN2 levels and a decrease in association of LDs with lysosomes (Schweiger and Zechner, 2015). These findings are consistent with the model that hsc70 binds to LD-associated PLIN2 and that CMA of PLIN2 occurs at MCS between LD and the lysosome (Figure 4IV).

CMA of PLIN2 and PLIN3 is triggered by stressors including nutrient limitation, oxidative and lipogenic stresses, and hypoxia (Cuervo et al., 1995; Kiffin et al., 2004; Dohi et al., 2012; Rodriguez-Navarro et al., 2012; Kaushik and Cuervo, 2015), and contributes to stressor-stimulated release of lipids from LDs. Specifically, removal of PLIN2 and PLIN3 from the LD surface promotes association of LDs with 1) cytosolic lipases (e.g., ATGL) that catalyze release of FA from TAG and 2) the LD macroautophagy machinery. In turn, this promotes the release of lipids from LDs after degradation by the lysosome (Kaushik and Cuervo, 2015). These findings support a function of LD-lysosome MCS, and a role for CMA in regulation of lipid homeostasis.

## 5 Cytoskeletal Modulation of LD-Organelle Interactions

As described above, environmental cues including nutrient availability and exposure to stressors induce MCS formation between LDs and organelles including mitochondria, ER and lysosomes. The cytoskeleton plays a fundamental role in this process by controlling the position and movement of LDs and organelles that interact with LDs. For example, in response to nutrient limitation, LDs change from clustered to a dispersed distribution, which allows LDs to make contact with mitochondria for up-regulation of lipid metabolism (Herms et al., 2015; Nguyen et al., 2017; Kong et al., 2020). Although multiple mechanisms have been identified for cytoskeletal control of organelle motility, the best characterized mechanism relies on motor molecule-driven, polarized movement of organelles along actin or microtubule tracks. Here, we summarize cytoskeletal function in LD interactions and contact site formation with other organelles.

### 5.1 Evidence of Cytoskeleton-Directed LD Distribution and Motility

Cytoskeletal components and motors have been found on LDs in a variety of organisms, including fungi, plants, and mammals. Proteomic analysis of LDs revealed actin, tubulin, and motor proteins on LDs (Turró et al., 2006; Weibel et al., 2012; Brocard et al., 2017; Pfisterer et al., 2017; Yu et al., 2017; Zhi et al., 2017; Bersuker et al., 2018). In particular, a high-confidence LD proteome generated from proximity labeling confirmed that actin, tubulin, and a kinesin family protein, KIF16B, are recovered with isolated LDs (Bersuker et al., 2018). Additionally, immunofluorescence staining in rat adrenocortical cells and adipocytes showed that beta-actin is present on the LD surface (Fong et al., 2001).

The actin and microtubule cytoskeletal networks and their associated motor proteins are involved in LD morphology and distribution within the cell. For example, destabilization of the actin cytoskeleton by treatment with either cytochalasin D (CytD) or latrunculin-A decreases the size of LDs in J774 macrophages (Weibel et al., 2012). Destabilizing microtubules by nocodazole treatment also decreases LD size (Boström et al., 2005; van Zutphen et al., 2014; Gu et al., 2019). Presumably, this change in size results from a change in the balance between the addition and removal of LD cargo, which occurs at specific MCS. Consistent with this idea, the position and dynamics of LDs are also dependent on the cytoskeleton. Destabilization of actin filaments prevents LD movement from the vegetal pole to the animal pole in zebrafish embryos (Dutta and Kumar Sinha, 2015). Post-translational modifications of tubulin affect LD motility and distribution. For example, during nutrient deficiency, detyrosinated tubulins accumulate and form networks that promote LD dispersion in Vero cells (Herms et al., 2015). In contrast, acetylated tubulins immobilize LDs in hepatic cells (Groebner et al., 2019).

**FIGURE 5:**
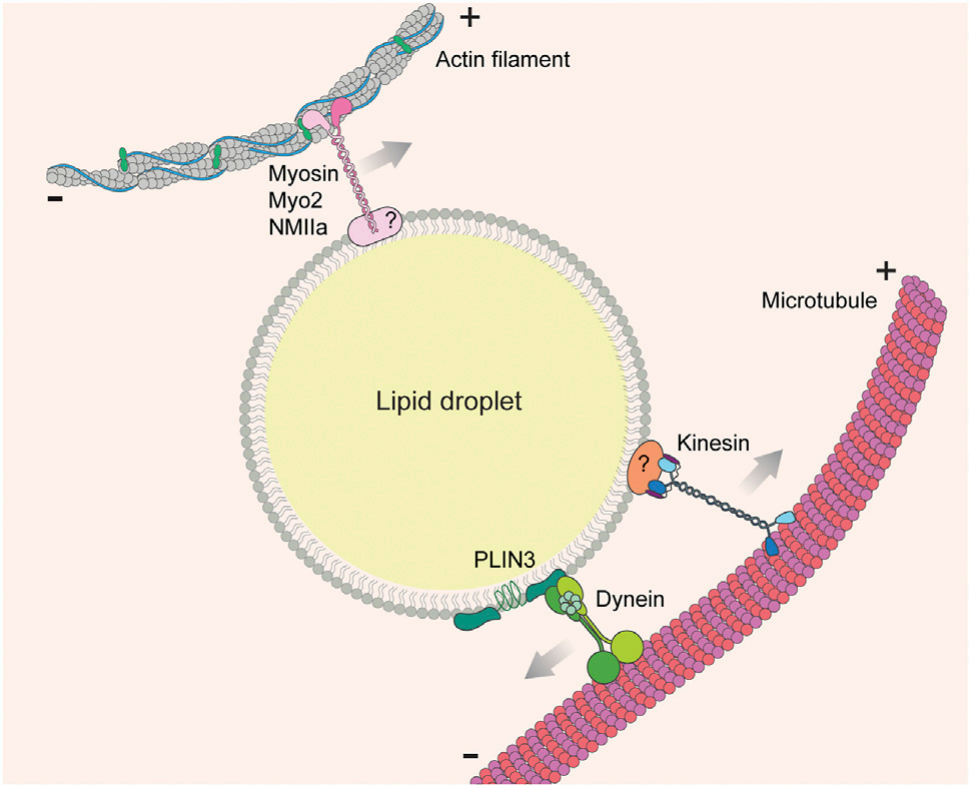
LD-cytoskeleton interaction. Lipid droplets are transported on cytoskeletal fibers (actin filaments or microtubules) by cytoskeleton-associated motor molecules (myosins, kinesins, and dyneins). In most cases the adaptors linking motors to the LD surface are unknown.

Although these studies reveal that morphology and distribution of LDs depend on the cytoskeleton, it is not always clear whether the effects observed upon global destabilization of microtubule or actin cytoskeletons are due to direct effects on LD-cytoskeleton interactions. However, the effects of disrupting motor proteins, which drive motility on cytoskeletal tracks, are less ambiguous. Both the actin-based motor myosin and the microtubule-based motors kinesin and dynein drive LD distribution and motility (Figure 5) (Gross et al., 2000; Andersson et al., 2006; Shubeita et al., 2008; Knoblach and Rachubinski, 2015; Pfisterer et al., 2017; Rai et al., 2017; Gu et al., 2019; Veerabagu et al., 2020).

In some cases, specific motor proteins that drive LD motility have been identified. In budding yeast, anterograde movement of LDs from mother cells to buds relies on a type V myosin, Myo2p (Figure 5) (Knoblach and Rachubinski, 2015). During zebrafish development, inhibiting Myosin-1 with pentachloropseudilin alters the dynamics and distribution of LDs (Gupta et al., 2017). Knockdown of non-muscle myosin IIa (NMIIa) enlarges LDs and promotes their clustering in human osteosarcoma U2OS cells (Pfisterer et al., 2017). Post-translational modification of motor proteins also alters motor-LD interactions. For example, ERK-mediated phosphorylation of dynein increases its affinity for LDs (Andersson et al., 2006). Motor knock-down studies are somewhat more specific than drug-induced cytoskeletal disruption, but still may be subject to pleiotropic effects because motor proteins are shared by multiple cargos. A more specific approach is to target the cargo adaptor proteins that bridge LDs and cytoskeletal/motor proteins, although these adaptors are less well understood. One recently identified adaptor is the LD protein perilipin 3 (PLIN3), which interacts with the dynein intermediate chain subunit, Dync1i1, in AML12 mouse hepatic cells (Figure 5) (Gu et al., 2019). Identifying more of these LD-specific cargo adaptor proteins will allow in-depth characterization of the biological function of LD-cytoskeletal interaction.

### 5.2 Functional Consequences of LD-Cytoskeleton Interactions

MCSs between LD and other organelles play an important role in exchanging metabolites. Therefore, any change in the distribution or dynamics of those sites can affect their function. Indeed, not only the size, but also the lipid composition of LDs in J774 macrophages is changed by actin destabilization (Weibel et al., 2012). Destabilization of the actin cytoskeleton reduces the dissociation of LDs from peroxisomes in *Arabidopsis* (Cui et al., 2016). Microtubules are required for LD autophagy (Boström et al., 2005; van Zutphen et al., 2014; Gu et al., 2019). Nocodazole-treated COS-7 cells have fewer contact sites between LDs and mitochondria or peroxisomes, as well as fewer ternary contacts between LDs, peroxisomes, and Golgi (Valm et al., 2017).

LD-mitochondria interactions are crucial for mobilizing the energy stored in LDs (Rambold et al., 2015). When nutrients are depleted, Vero cells exhibit dispersion of LDs, and a concomitant increase in LD-mitochondria contacts, consistent with the need for lipid exchange and fatty acid metabolism. Starvation-induced LD-mitochondria contacts include both relatively short-lived interactions (“touch and go”) and more stable connections (Herms et al., 2015). Microtubules are required for formation of these contacts (Valm et al., 2017), and the dispersion of LDs from the perinuclear area to the cell periphery specifically depends on detyrosinated microtubules. Detyrosination is promoted by the activation of the energy sensor, AMP protein kinase (AMPK) (Herms et al., 2015). AMPK also phosphorylates PLIN3, which may induce conformational changes of PLIN3 to facilitate LD dispersion (Zhu et al., 2019). Given the link between PLIN3 and dynein and microtubules (Gu et al., 2019), the LD dispersion caused by phosphorylated PLIN3 may be due to the altered interaction between PLIN3 and dynein. Taken together, LD-mitochondria interactions are elevated upon starvation, and this response requires the microtubule network to shuttle LDs to mitochondria and facilitate lipid metabolism in this system.

In another well-characterized system, microtubule-based motor proteins on the surface of LDs stimulate lipid transfer to ER and therefore facilitate lipoprotein assembly in liver cells (Rai et al., 2017). In rat hepatocytes, LDs are actively transported by the motor molecule kinesin-1 on microtubules to the cell periphery, which promotes MCS formation between LDs and smooth endoplasmic reticulum (sER) (Barak et al., 2013; Rai et al., 2017; Kumar et al., 2019). Kinesin-1 is recruited to LDs by directly binding to phosphatidic acid (PA) (Kumar et al., 2019). However, this binding is dependent on the metabolic state of the cells. In nutrient-rich conditions, the GTPase ADP ribosylation factor 1 (ARF1) recruits PA-producing phospholipase-D1 (PLD1) to LDs, which results in elevation of PA levels on LDs, increased association of kinesin-1 with LDs (Wilfling et al., 2014; Rai et al., 2017; Kumar et al., 2019). These LDs are then actively transported to cell periphery to form MCS with sER, which facilitates TAG production in sER and very low density lipoprotein (VLDL) assembly (Thiam et al., 2013; Rai et al., 2017; Kumar et al., 2019). In contrast, in the fasted state, insulin levels decrease, which downregulates the recruitment of ARF1 to the LDs. This diminishes microtubule-dependent LD movement and the formation of LD-sER MCSs at the periphery, resulting in reduced TAG levels (Kumar et al., 2019).

Dynein and microtubules are also involved in LD biogenesis in the alcohol-induced liver damage model (Gu et al., 2019). High-alcohol diets induce accumulation of LDs and elevate the levels of perilipins in liver cells, including the dynein-interacting protein PLIN3. Moreover, immunofluorescence staining revealed that Dync1i1 colocalizes with LDs, and PLIN3 and LDs are partially colocalized with microtubules. Depolymerizing microtubules by nocodazole or knocking down PLIN3 inhibits LD biogenesis from LD-ER contact sites, which reduces the size and distribution of LDs in AML12 cells.

The examples discussed above illustrate the importance of cytoskeletal function in regulating interactions between LDs and other organelles. Cellular modulation of the number and dynamics of these MCS is vital for LD biogenesis, lipid secretion and lipoprotein assembly.

## 6 Conclusion and Future Directions

MCS that form between LDs and organelles including mitochondria, ER and lysosomes/vacuoles function in LD biogenesis and in transfer of lipids, FAs, unfolded proteins and surplus or toxic proteins to or from LDs. Moreover, emerging evidence supports a role for the cytoskeleton in formation of MCS between LDs and other organelles by controlling the position and movement of LDs in response to environmental cues. However, fundamental questions regarding LD MCS remain unanswered. While many tethers that link LDs to mitochondria under conditions of nutrient limitations have been identified, the mechanisms that regulate LD-mitochondria MCS formation and loss are not well understood. Although LD-ER contact sites have an established function in LD biogenesis, the mechanism underlying scission of nascent LDs from ER membranes at LD-ER MCS is not known. The finding that LDs function in ER proteostasis through transfer of unfolded proteins from ER to LDs at LD-ER MCS revealed a novel function for LDs. However, it is not clear whether this process is linked to LD biogenesis. Indeed, if mature LDs can associate with LDs to remove unfolded proteins and mitigate ER stress, the proteins serve as tethers at those LD-ER MCS and mechanisms that promote those MCS remain unknown. Moreover, the proteins that tether LDs to lysosomes/vacuoles; how liquid ordered (L_o_) and disordered (L_d_) domains in the vacuolar membrane contribute to MCS and vacuolar membrane dynamics at those sites, and the mechanism underlying Rab7 function in LD-lysosome/vacuole MCS in mammalian cells and yeast during µLP are all open questions. Finally, while it is clear that cytoskeleton-dependent LD motility is critical for association of LDs with other organelles in response to nutritional cues, the cytoskeleton may contribute to MCS by other mechanisms including force generation for membrane deformation or scission or for transfer of constituents to and from LDs.

## Abbreviations

ATGL, adipose triglyceride lipase; CMA, chaperone-mediated autophagy; ERAD, ER-associated degradation pathway; ESCRT, endosome sorting complex required for transport; HH, hydrophobic helix; LTD, lipid transfer domain; µLP, macrolipophagy; MCS, membrane contact sites; MVB, multivesicular bodies; VAB, VPS13 adaptor binding.
